# Inhibition of EPAC1 signaling pathway alters atrial electrophysiology and prevents atrial fibrillation

**DOI:** 10.3389/fphys.2023.1120336

**Published:** 2023-02-22

**Authors:** Bastien Guillot, Arthur Boileve, Richard Walton, Alexandre Harfoush, Caroline Conte, Yannis Sainte-Marie, Sabine Charron, Olivier Bernus, Alice Recalde, Laurent Sallé, Fabien Brette, Frank Lezoualc’h

**Affiliations:** ^1^ IHU LIRYC -CRCTB U1045, Pessac, France; ^2^ INSERM U1045 -Université de Bordeaux, Bordeaux, France; ^3^ UR 4650 PSIR, GIP Cyceron, Caen, France; ^4^ Université de Caen-Normandie, Caen, France; ^5^ Université de Toulouse-Paul Sabatier, Toulouse, France; ^6^ Institut des maladies métaboliques et cardiovasculaires, INSERM UMR-1297, Toulouse, France; ^7^ PhyMedExp, INSERM U1046, CNRS 9412, Université de Montpellier, Montpellier, France

**Keywords:** EPAC, atrial fibrillation, optical mapping, cardiomyocytes, action potential

## Abstract

**Introduction:**

Atrial fibrillation (AF) is the most common sustained cardiac arrhythmia and is associated with increased mortality and morbidity. The Exchange Protein directly Activated by cAMP (EPAC), has been implicated in pro-arrhythmic signaling pathways in the atria, but the underlying mechanisms remain unknown.

**Methods:**

In this study, we investigated the involvement of EPAC1 and EPAC2 isoforms in the genesis of AF in wild type (WT) mice and knockout (KO) mice for EPAC1 or EPAC2. We also employed EPAC pharmacological modulators to selectively activate EPAC proteins (8-CPT-AM; 10 μM), or inhibit either EPAC1 (AM-001; 20 μM) or EPAC2 (ESI-05; 25 μM). Transesophageal stimulation was used to characterize the induction of AF *in vivo* in mice. Optical mapping experiments were performed on isolated mouse atria and cellular electrophysiology was examined by whole-cell patch-clamp technique.

**Results:**

In wild type mice, we found 8-CPT-AM slightly increased AF susceptibility and that this was blocked by the EPAC1 inhibitor AM-001 but not the EPAC2 inhibitor ESI-05. Consistent with this, in EPAC1 KO mice, occurrence of AF was observed in 3/12 (vs. 4/10 WT littermates) and 4/10 in EPAC2 KO (vs. 5/10 WT littermates). In wild type animals, optical mapping experiments revealed that 8-CPT-AM perfusion increased action potential duration even in the presence of AM-001 or ESI-05. Interestingly, 8-CPT-AM perfusion decreased conduction velocity, an effect blunted by AM-001 but not ESI-05. Patch-clamp experiments demonstrated action potential prolongation after 8-CPT-AM perfusion in both wild type and EPAC1 KO mice and this effect was partially prevented by AM-001 in WT.

**Conclusion:**

Together, these results indicate that EPAC1 and EPAC2 signaling pathways differentially alter atrial electrophysiology but only the EPAC1 isoform is involved in the genesis of AF. Selective blockade of EPAC1 with AM-001 prevents AF in mice.

## 1 Introduction

Atrial fibrillation (AF) is the most common sustained cardiac arrhythmia, associated with ≈2 fold increased risk of mortality and is predicted to affect 2.5 times more people in industrialized countries in the next 50 years, with a 3% increase in prevalence amongst young adults (>20 years) (Chugh et al., 2014; Kirchhof et al., 2016). AF is a progressive arrhythmia naturally evolving from transient self-terminating episodes into chronic episodes (paroxysmal to persistent AF) increasing susceptibility to major complications such as stroke, heart failure and dementia. This disease is characterized by chaotic electrical activity and a loss of mechanical contraction in the atria due to arrhythmogenic areas localized to the roots of pulmonary veins in the left atria in >90% of patients with paroxysmal AF (Haissaguerre et al., 1998). Treatments currently available are mainly efficient in paroxysmal AF and very inconclusive in persistent AF, when arrhythmia is longer and the substrate more complex. Several mechanisms are known to be involved in AF development and sustainability such as: electrical remodeling caused by ion channel expression dysregulation, action potential (AP) duration shortening, abnormal Ca^2+^ cycling inducing modifications of excitation-contraction coupling (EC coupling) related to cardiomyocyte contraction, and structural remodeling involving fibrosis responsible for alterations in electrical conduction through the myocardium (Nattel, 2017). There are however still gaps in our knowledge on processes related to the pathophysiology of AF (Heijman et al., 2014; Heijman et al., 2018).

The second messenger cyclic AMP (cAMP) regulates diverse physiologic processes including Ca^2+^ homeostasis, cellular permeability, and gene expression. The major intracellular functions of cAMP are transduced by protein kinase A (PKA) and by the more recently identified cAMP binding proteins, EPACs. The two EPAC isoforms, EPAC1 and EPAC2, act as guanine-nucleotide exchange factors for the small G protein, Rap and function in a PKA-independent manner (Lezoualc’h et al., 2016; Bouvet et al., 2019). In ventricular cardiomyocytes, EPAC activation modulates EC coupling. However, EPAC does not play a major role in the inotropic response to acute β-adrenergic receptor (β-AR) stimulation compared with PKA, which is the main cAMP effector in this process (Pereira et al., 2013; Laurent et al., 2015). Acute activation of EPAC with the cAMP analogue 8-CPT enhanced pro-arrhythmia mechanisms, including spontaneous diastolic Ca^2+^ leak *via* a hyperphosphorylation of the ryanodine receptor (RyR) and increased action potential duration (APD) in isolated ventricular cardiomyocytes from rat (Pereira et al., 2007; Brette et al., 2013). The arrhythmogenic effect of EPAC activation was confirmed in Langendorff-perfused and isolated murine hearts (Hothi et al., 2008; Li et al., 2017).

Strong evidence suggests that EPAC is involved in ventricular cardiac arrhythmia in mice (Bouvet et al., 2019). However, there is a paucity of study in the atria, especially in the context of AF. Two isoforms of EPAC, EPAC1 and EPAC2, are expressed in the heart. A recent study has identified that EPAC1 inhibition could reduce AF occurrence in a mouse model of heart failure (Zhang et al., 2019). Similarly, a second study indicated that EPAC1 is involved in the development of atrial and ventricular arrhythmias in mice (Prajapati et al., 2019). Thus, pharmacological modulation of EPAC is of interest and recently, a thieno[2.3-b]pyridine derivative, designated as AM-001, has been developed as a selective EPAC1 pharmacological inhibitor (Laudette et al., 2019).

Therefore, to delineate the role of EPAC1 and EPAC2 isoforms in atrial electrophysiology and AF susceptibility, we employed mouse models that were deleted for EPAC1 (EPAC1^−/−^), EPAC2 (EPAC2^−/−^) and a pharmacological approach.

## 2 Material and methods

### 2.1 Ethics

The animal study was reviewed and approved by the ethical committee of Bordeaux, Caen and Toulouse, France. All experiments were conducted in accordance with the European Union directive EU/2010/63.

### 2.2 Animals

6- to 10-week-old EPAC WT (C57Bl/6), EPAC1^−/−^, and EPAC2^−/−^ male mice were used in this study. Global knock-out for EPAC1 isoform has been described previously (Laurent et al., 2015).

### 2.3 Generation of EPAC2^−/−^ mice

RAPGEF4 gene was floxed with two LoxP sites around Ex 17 by Institut Clinique de la Souris (IGBMC, Strasbourg) using homologous recombination in C57/Bl6N ES cells. The Myh6-Cre mice with a C57/Bl6J genetic background expressing Cre recombinase in cardiomyocytes were purchased from Janvier labs (France). Bl6N have a functional, wild-type Nicotinamide Nucleotide Transhydrogenase (NNT) while Bl6J have a defective allele and loss of enzymatic activity has been associated with mitigated heart failure upon pressure overload (Nickel et al., 2015). Heterozygous EPAC2 flox and Myh6-Cre mice were first crossed and their progeny were selected to obtain these genetic modifications along with an homozygous wild-type allele at NNT locus. Then, Myh6-Cre (+/−) EPAC2 (flox/wt) were crossed with Myh6-Cre (−/−) EPAC2 (flox/wt) and progeny was used for breeding to generate Myh6-Cre(+/−) EPAC2 (flox/flox) and Myh6-Cre (+/−) EPAC2 (wt/wt) which were used as control.

The following primers are used for genotype analysis. For Nnt locus, a set of three primers: common (5′-GTA​GGG​CCA​ACT​GTT​TCT​GCA​TGA-3′),

Wt (5′-GGG​CAT​AGG​AAG​CAA​ATA​CCA​AGT​TG-3’)

Mutant (5′- GTG​GAA​TTC​CGC​TGA​GAG​AAC​TCT​T-3′).

For Cre recombinase:

(5′-CCT​GGA​AAA​TGC​TTC​TGT​CCG-3′) and (5′-CAG​GGT​GTT​ATC​CGC​AAT​CCC-3′).

For EPAC2flox:

(5′-CCT​GTG​TGC​GTA​GGT​GTC​TTC​GTC-3‘) and (5'-GGC​ATT​TGT​AAC​CTT​CAA​AAC​ACA​TG-3')

Supplementary Figure S1 shows quantitative RT-PCR results.

### 2.4 Echocardiography

Mice were anesthetized with isoflurane (3% induction and 1%–1.5% maintenance), placed on a heating pad and examined by echocardiography (Vevo2100, VisualSonics). M-mode in parasternal short axis view at the level of papillary muscle was used for wall thickness (LVAW, LVPW), cavity diameter (LVID) and fractional shortening (FS) measurements. 2D-mode long axis view was used for ejection fraction (EF). Images were analysed off-line with VevoLab software (VisulaSonics). Acquisition and analysis were performed blind.

Supplementary Table S1 shows that EPAC2^−/−^ are not significantly different from WT for heart size and cardiac function except for a small decrease in heart rate and left ventricle end-diastolic posterior wall thickness.

### 2.5 Drug treatment

All solutions were prepared using ultrapure water supplied by a Milli-Q system (Millipore, USA). All of the reagents used in this study were purchased from Sigma Aldrich (France) unless otherwise stated. Liberase was purchased form Roche (France). The acetoxymethyl ester form of 8-CPT (8-CPT-AM) and the EPAC2 inhibitor were purchased from Biolog Life Science Institute (Germany). AM-001 was purchased from Abinter (France). To activate EPAC pathway, 8-CPT-AM was used at 10 μM. AM-001 was used at 20 μM and ESI-05 at 25 μM to selectively inhibit EPAC1 or EPAC2 isoform, respectively.

### 2.6 Reverse transcription quantitative PCR (RT-qPCR)

Atria from seven mice were dissected and immediately placed into All protect tissue solution (Qiagen, Germany). Atria were then stored at −80°C until RNA extraction. Total RNA was extracted from atria and purified using the QIAzol reagent (Qiagen) and the Qiagen RNeasy Kit (Qiagen). RNA quantity was then measured by spectrophotometry using the NanoDrop (Thermofisher, USA). 100 ng of RNA was reversed transcribed using cDNA Reverse Transcription kit (BIO-RAD). Primers were synthesized and obtained from BIO-RAD. RT-qPCR was performed in a 10 μL reaction volume composed by cDNA, SYBR Green mix (BIO-RAD, USA), ddH2O and primers on the BIO-RAD C100 Touch Thermal Cycler/CFX96 Real-time System. Expression levels for RAPGEF3 (EPAC1) and RAPGEF4 (EPAC2) were normalized using CFX Manager software (BIO-RAD) and housekeeping genes, GAPDH and HPRT1.

### 2.7 Transesophageal atrial stimulation

AF inducibility and probability were determined by transesophageal atrial stimulation (TS). Mice atria were stimulated using an octopolar catheter (WPI) introduced in the oesophagus under 2% isoflurane (Supplementary Figure S2A). Right atrial stimulation consisted of a burst pacing protocol of 40 stimuli at each pacing cycle with decreasing cycle length from 60 to 10 ms by 2 ms steps (Supplementary Figure S2B). Two forelimb electrodes were used to record ECG. Analysis was performed with Spike 2 software (Cambridge Electronic Design). AF was defined as an irregular and rapid rhythm lasting for more than 2 s and associated with loss of P wave (Verheule et al., 2004) (Supplementary Figure S2C). A mouse is considered as inducible when an AF episode was identified after burst pacing. AF probability (%) is defined by the ratio of the number of AF episodes divided by the number of burst pacing. The treatment of mice with the pharmacological activator and inhibitors of EPAC pathway or vehicle (0.1% dimethyl sulfoxide [DMSO]) was administered 30 min before the induction of AF *via* intraperitoneal injection.

### 2.8 High resolution optical mapping

Mice were anesthetized by inhalation of isoflurane (2% in air) and then humanly killed by cervical dislocation. Heart was quickly removed and atria were isolated in cardioplegic solution (in mmol/L: 110 NaCl, 1.2 CaCl_2_, 16 KCl, 16 MgCl_2_, 10 NaHCO_3_, 9 Glucose). Then atria were pinned down on a Sylgar gel in the superfusion chamber before being perfused with Tyrode solution (in mmol/L: 130 NaCl, 24 NaHCO_3_, 1.2 NaH_2_PO_4_, 1 MgCl_2_, 5.6 Glucose, 4 KCl, 1.8 CaCl_2_) gassed with 5% CO2 and 95% O2 (pH 7.4) at 37°C ± 0.5°C (Supplementary Figure S3A). Optical mapping experiments were performed using a high resolution CMOS camera (MICAM Ultima, SciMedia, USA) recording from a 100 x 100 field of view, at an 0.11 cm isotropic spatial resolution and a temporal resolution of 1 kHz. Right atria were paced with two unipolar electrodes at 2 ± 0.5 mA at frequencies ranging from 7 Hz to 12 Hz. Voltage-sensitive optical signals were obtained using di-4-ANEPPS (10 µM, VWR International) excited at 530 nm with LEDs (Cairn Research Ltd, Kent—United Kingdom). The fluorescence light was filtered with a band pass filter at 650 nm. Optical AP were analyzed using the PVwave software to quantify AP duration (APD) and calculate conduction velocities (CV). The effects of activation of the pathways for EPAC1 (8-CPT+ESI-05) and EPAC2 (8-CPT+AM-001) were evaluated by superfusion.

### 2.9 Mouse atrial myocytes isolation

Atrial Myocytes were dissociated as previously described (Jansen and Rose, 2019). Briefly, mice were anesthetized by inhalation of isoflurane (2% in air) then heparinized by intraperitoneal injection of Heparin (200 UI). Mice anesthesia was checked by absence of the paw withdrawal reflex. Mice were subsequently killed by cervical dislocation and atrial appendages were rapidly excised. After the excision, all digestion steps were realized at 37°C. Atria were quickly washed and minced in modified Tyrode solution (in mmol/L: 140 NaCl, 5.4 KCl, 1.2 KH_2_PO_4_, 5 HEPES, 5.55 Glucose, 1 MgCl_2_, 1.8 CaCl_2_, 5 U/mL Heparin; pH 7.4 with NaOH) and transferred in a pre-digestion buffer solution (in mmol/L: 140 NaCl, 5.4 KCl, 1.2 KH_2_PO_4_, 5 HEPES, 18.5 Glucose, 50 Taurine, 0.066 CaCl_2_, 1 mg/mL Bovine Serum Albumin; pH 6.9 with NaOH). After 5 min of pre-digestion, tissues were transferred in a digestion solution corresponding to the pre-digestion buffer supplemented by 0.11 mg/mL (equivalent to 0.34 Wünsch unit/mL and 36.7 units/mL Dispase) of Liberase (Medium Thermolysine, Roche, France). The digestion step lasted 20–23 min. After digestion was completed, atrial stripes were washed in a modified Kraft-Brühe solution (in mmol/L: 100 K-Glutamate, 10 K-Aspartate, 25 KCl, 10 KH_2_PO_4_, 2 MgSO4, 20 Taurine, 5 Creatine, 0.5 EGTA, 20 Glucose, 5 HEPES, 0,1% Bovine Serum Albumin; pH 7.2 with KOH), and mechanically triturated in this solution to allow cell isolation. Once the dissociation ended, cells were gradually reintroduced to 1 mmol/L calcium concentration by addition of calcium in the Kraft-Brühe solution (in mmol/L of free calcium: 0.125, 0.25, 0.375, 0.5, 0.625, 0.75, 0.875, and 1). Cells were used for patch clamp experiments during the 8 h following the dissociation. Only rod shaped and striated cells were used for experiments.

### 2.10 Cellular electrophysiological recording

Isolated myocytes were studied in Petri dishes on the stage of an inverted microscope (Nikon TE200-S, Japan). AP were recorded at room temperature using the whole cell configuration of the patch-clamp technique in its current-clamp mode. For data acquisition, an Axopatch 200B (Molecular Devices, United State) amplifier connected to a Digidata 1322 A/D (Molecular Devices, United State) were used. Data were recorded and analyzed using pClamp software 9 (Molecular Devices, United State). Signals were digitized at a frequency of 10 KHz and filtered at 2 KHz using a 8-pole Bessel low pass filter. Patch pipettes resistance was usually comprised between 1.2 and 2.5 MΩ when filled with the intrapipette solution described below.

AP were elicited by 1 ms supra-threshold current steps at a frequency of 0.1 Hz. Bath solution was composed by (in mmol/L): 130 NaCl, 5.4 KCl, 1.4 MgCl_2_, 0.4 NaH_2_PO_4_, 4.2 HEPES, 10 Glucose, 20 Taurine, 10 Creatine, 1 CaCl2; pH 7.4 with NaOH. Pipette solution was composed by (in mmol/L): 10 NaCl, 130 K-Glutamate, 9 KCl, 5 ATPMg, 0.5 MgCl_2_, 10 HEPES, 0.4 GTP-Tris, 0.5 EGTA, 0.12 CaCl_2_; pH 7.2 with KOH.

AP amplitude was measured as the difference between the peak of overshoot and the resting membrane potential. The maximum rate of rise of the AP (dV/dtmax) was calculated by differentiation of the AP upstroke using Clampfit software. Action potential duration (APD) was measured as the duration from the trigger of AP to 20%, 50% and 90% of repolarization (APD20, APD50 and APD90, respectively).

AP parameters under 8-CPT-AM superfusion (10 μmol/L) have been assessed at the steady state effect of the compound (∼5 min). To evaluate the EPAC1 selective inhibition by AM-001, cells were first treated by 8-CPT-AM (10 μmol/L) alone, then co-treated for at least 15 min by superfusion of both 8-CPT-AM (10 μmol/L) and AM-001 (20 μmol/L). The impact of the co-treatment has been evaluated at the steady state of the effect after this time lapse.

### 2.11 Statistical analysis

Data are reported as mean ± SEM. Statistical analysis was performed using GraphPad Prism 8 software. Data were tested for normal distribution using Shapiro-Wilk test. Fisher’s exact tests were used to evaluate AF inducibility. For optical mapping data, Student’s paired and unpaired *t*-test and non-parametric test (Mann-Whitney) were used to compare two populations, and ANOVA or Kruskal Wallis tests with appropriates *post hoc* tests (Tukey’s and Dunn’s test) were used to evaluate differences between more than two groups. For patch clamp data, two-way repeated measure ANOVA with Holm-Sidak *post hoc* test for normality distributed, otherwise Friedman’s ANOVA on ranks with Dunn’s multiple comparisons *post hoc* were used. *p* < 0.05 was considered statistically significant.

## 3 Results

### 3.1 EPAC1 deficiency decreased AF inducibility

**FIGURE 1:**
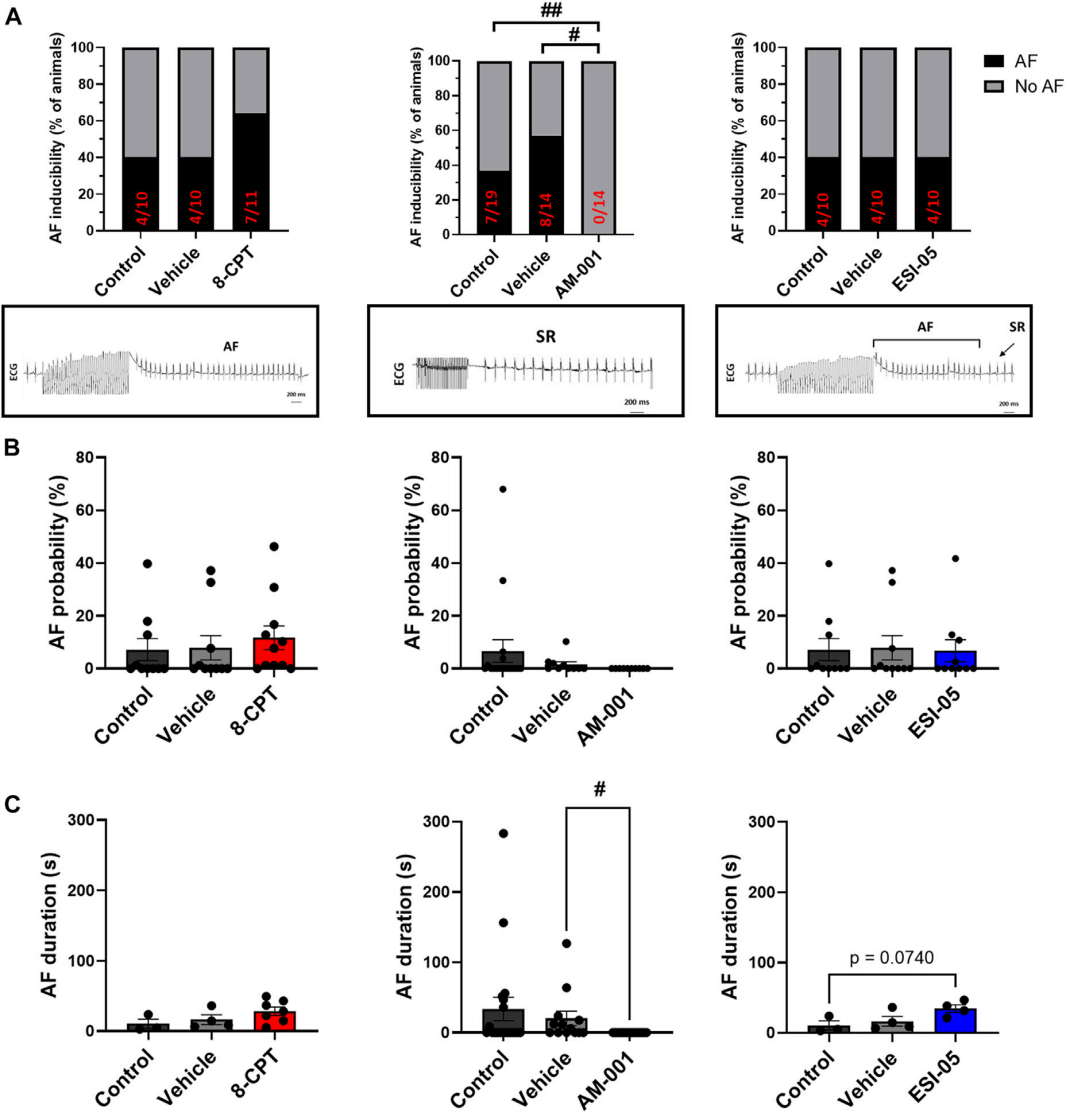
*In vivo* evaluation of AF inducibility, probability and duration by modulation of EPAC activity in WT mice. **(A)** Number of animals in percent (%) with AF inducibility under EPAC1&2 activation (*N* = 11), EPAC1 inhibition (*N* = 14) and EPAC2 inhibition (*N* = 10). Insets below show representative ECG recordings. **(B)** AF probability (%) after EPAC1&2 activation and EPAC1 and EPAC2 inhibition. **(C)** Mean ± SEM of AF duration before and after pharmacologic modulations of EPAC1 and EPAC2 activation and inhibition. Statistical differences were determined by Fisher’s exact tests **(A)**, and by ANOVA with Tukey post hoc test and Kruskal Wallis with Dunn *post hoc test* [**(B,C)**, #*p* < 0.05; ##*p* < 0.01].

We first assessed the expression of EPAC1 and EPAC2 mRNA in mouse atria. Consistent with a previous study (Zhang et al., 2019), both isoforms are present (Supplementary Figure S4). Next, we determined the role of EPAC1 and EPAC2 in AF susceptibility *in vivo* using a pharmacological approach. Activation of EPAC1 and EPAC2 by injection of 8-CPT-AM slightly increased the proportion of animals showing AF episodes (Figure 1A). Indeed, AF was recorded only in 40% of WT mice (4/10) and after injection of vehicle (4/10) while it was detected in 64% of mice after 8-CPT injection (7/11). Inhibition of EPAC1 by intraperitoneal injection of AM-001 completely prevented AF initiation (0% vs. 37% in control). In contrast, blocking EPAC2 with ESI-05 did not change AF inducibility compared to control and vehicle (40% of mice in each group). Figure 1B showed that there is trend in an increase of AF probability after injection of 8-CPT injection (7.18% ± 4.15% vs. 7.88% ± 4.58% vs. 11.66% ± 4.17%, control vehicle, 8-CPT respectively). AF probability was completely suppressed following AM-001 injection (EPAC1 inhibition) whereas inhibition of EPAC2 with ESI-05 did not alter AF probability (7.18% ± 4.15% vs. 7.88% ± 4.58% vs. 6.79% ± 4.17%, control vehicle, ESI-05 respectively). Interestingly, we found that there was a tendency for longer AF episodes under EPAC1 and EPAC2 activation by 8-CPT-AM treatment (10.46 ± 6.65 vs. 16.45 ± 6.8 vs. 28.55 ± 5.95 s, control, vehicle, 8CPT respectively) and with EPAC2 inhibition (10.46 ± 6.65 vs. 16.45 ± 6.8 vs. 34.37 ± 5.38 s, control, vehicle, ESI-05 respectively) (Figure 1C). These data show that EPAC1 and not EPAC2 contribute to AF genesis.

**FIGURE 2:**
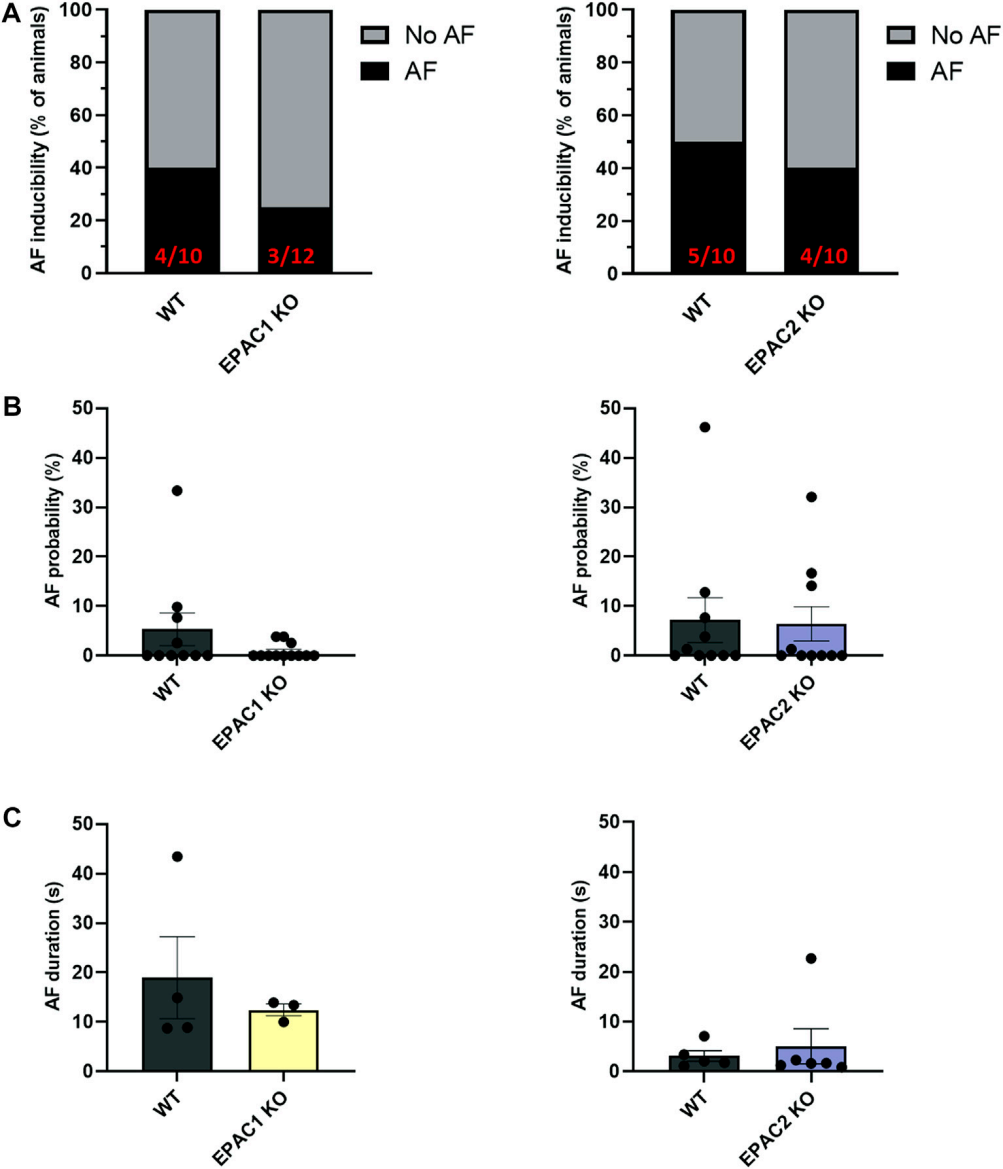
Impact of EPAC1 and EPAC2 expressions on AF. **(A)** AF vulnerability. **(B)** AF probability. **(C)** AF episode duration. Statistical differences were determined by Fisher’s exact tests **(A)** and Mann Whitney **(B,C)**; *N* = 10 WT, 12 EPAC1 KO, 12 EPAC2 KO.

To investigate further the role of EPAC1 in AF inducibility, we next used wild type (WT), EPAC1 KO and EPAC2 KO mice. Figure 2A shows that in EPAC1 KO mice occurrence of AF was slightly lower (3/12) than WT littermate (4/10). In contrast, in EPAC2 KO mice occurrence of AF was similar (4/10) than WT littermate (5/10). Furthermore, genetic deletion of EPAC1 decreased AF probability (5.34% ± 3.31% vs. 0.85% ± 0.45%) although it did not reach statistical significance (Figure 2B and see 4.5 Study limitations). No difference in AF probability was observed in EPAC2 KO mice compared to WT mice (7.18% ± 4.54% vs. 6.41% ± 3.49%). Lastly, there was tendency for a decrease in AF duration in EPAC1 KO mice (18.98 ± 8.27 s vs. 12.44 ± 1.21 s, WT and EPAC1 KO respectively) (Figure 2C).

Thus, it appears that EPAC1, but not EPAC2, is involved in atrial arrhythmia genesis. Subsequent experiments were then performed to characterize the role of EPAC1 and EPAC2 in atrial electrophysiology at tissue and cellular levels.

### 3.2 Atrial electrophysiology modulation by EPAC pathway

**FIGURE 3:**
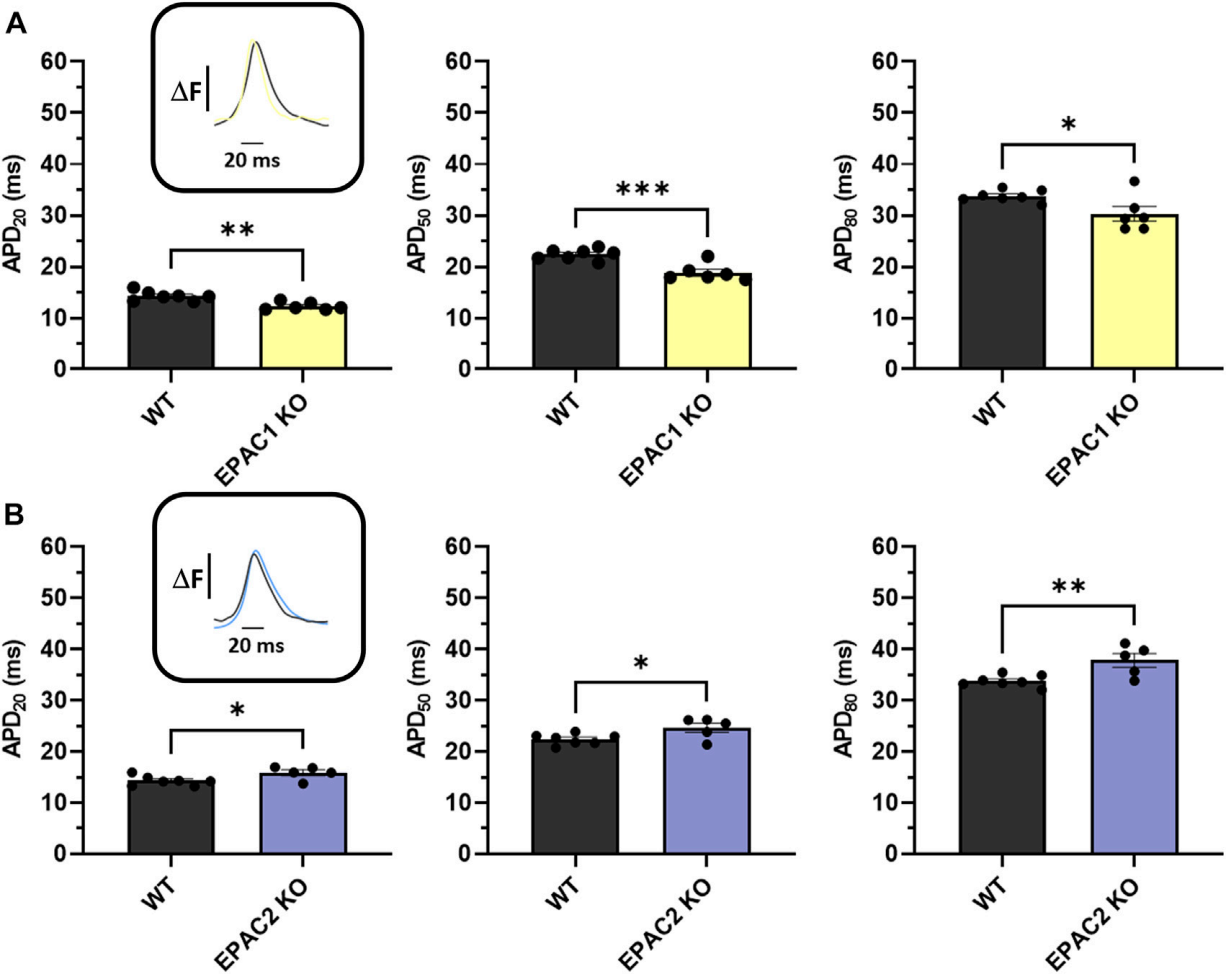
*Ex vivo* measurements of APD of WT and KO mice using optical mapping during 7 Hz pacing. **(A)** Means ± SEM of APD from LA and RA surfaces on WT (*N* = 7) and EPAC1 KO mice (*N* = 6). **(B)** Means ± SEM of APD from LA and RA surfaces on WT and EPAC2 KO mice (*N* = 5). Statistical differences were determined by unpaired t-tests (*p < 0.05; **p < 0.01; ***p < 0.001).

Next, we used high-resolution optical mapping to record optical AP in mice. Genetic deletion of EPAC1 significantly shortened APD20, APD50 and APD80 compared to WT mice (Figure 3A). Indeed, APD20 was decreased by 14% in EPAC1 KO mice (14.28 ± 0.35 m vs. 12.3 ± 0.30 m), APD50 was decreased by 16% (22.39 ± 0.39 vs. 18.86 ± 0.67 m), and APD80 by 10% (33.77 ± 0.43 vs. 30.30 ± 1.41 m). Conversely, genetic inhibition of EPAC2 significantly prolonged APDs (Figure 3B) since APD20, APD50, and APD80 were increased by 11% (14.28 ± 0.35 m vs. 15.86 ± 0.57 m), 10% (22.39 ± 0.39 vs. 24.60 ± 0.91 m), or 12% (33.77 ± 0.43 vs. 37.81 ± 1.34 m) in EPAC2 KO mice, respectively.

**FIGURE 4:**
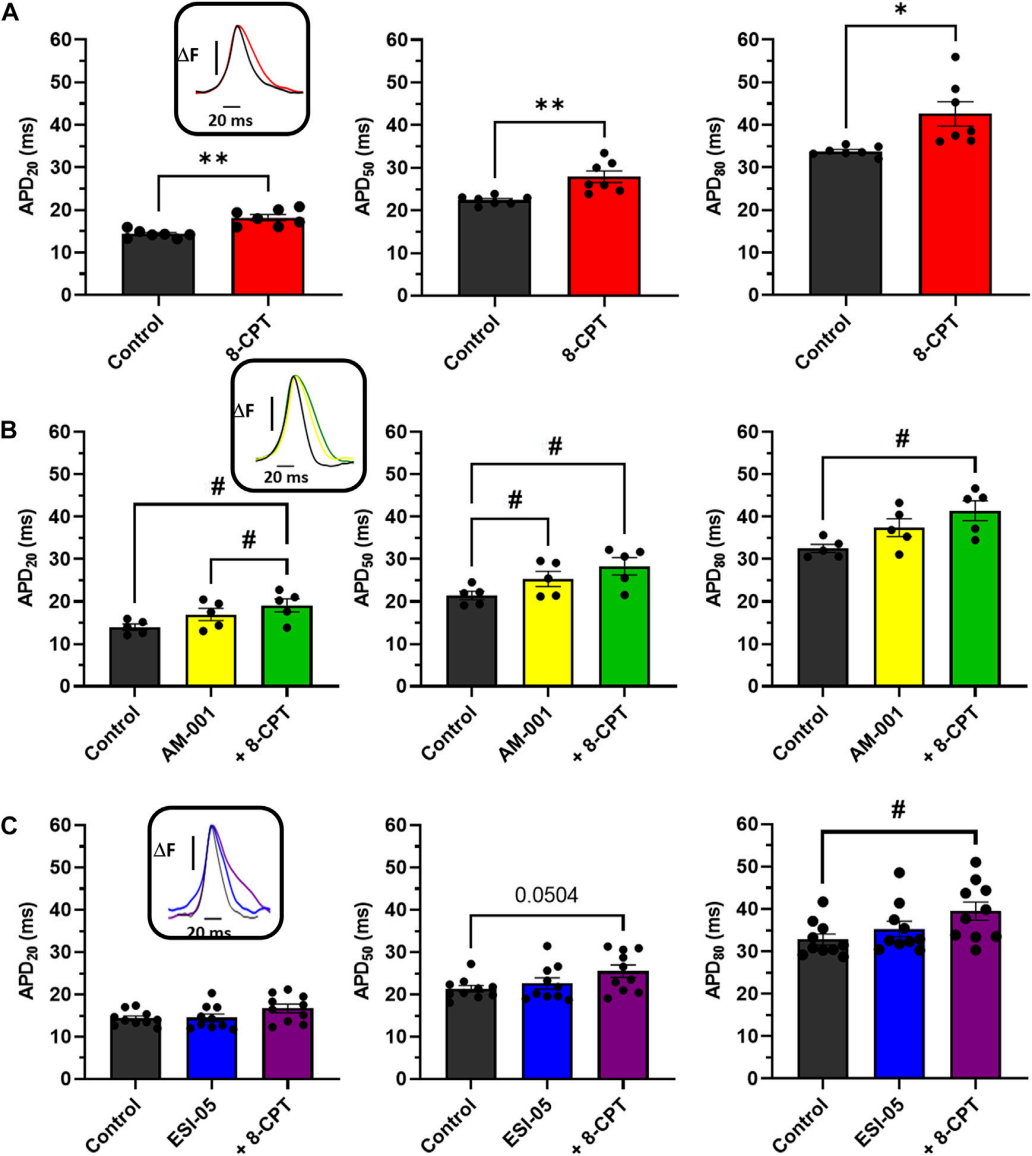
Impact of various EPAC isoforms on LA and RA APD. **(A)** Activation of EPAC1&2 by 8-CPT increases APD20, 50, 80. (*N* = 7) **(B)** Activation of EPAC2 pathway by inhibition of EPAC1 following by the activation of EPAC2 using AM-001 and 8-CPT increases APD20, 50, 80. (*N* = 5) **(C)** Activation of EPAC1 pathway by inhibition of EPAC2 following by the activation of EPAC1 using ESI-05 and 8-CPT tends to increase APD20, 50 and 8-CPT increases APD80. (*N* = 10). Statistical differences were determined by paired t-tests [**(A)**, *p < 0.05; **p < 0.01], and by ANOVA with Tukey *post hoc* test and Kruskal Wallis with Dunn *post hoc* test [**(B,C)**, #*p* < 0.05].

To further characterize the involvement of EPAC1 and EPAC2 in atrial electrophysiology, we used EPAC pharmacological probes approach. As reported in Figure 4A, 8-CPT-AM induced an increase of 27% of the APD20 (14.28 ± 0.35 m vs. 18.19 ± 0.72 m), an increase of 24% of the APD50 (22.39 ± 0.39 m vs. 27.86 ± 1.37 m), and of 26% of the APD80 (33.77 ± 0.43 vs. 42.58 ± 2.87 m). Next, we investigated which EPAC isoform was involved in this prolongation of the APD. Inhibition of EPAC1 with AM-001 did not change APD (Figure 4B). Adding 8-CPT-AM to activate EPAC2 in AM-001 treated mice induced significant prolongation by 37% of APD20 (13.95 ± 0.73 vs. 19.08 ± 1.54 m), by 32% of APD50 (21.40 ± 1.00 vs. 28.30 ± 2.06 m), and by 27% of APD80 (32.47 ± 0.97 vs. 41.40 ± 2.35 m). We did not observe any significant change in APD in ESI-05 treated mice whereas the use of 8-CPT-AM in the presence of ESI-05 to preferentially activate EPAC1 protein, significantly prolonged APD80 by 20% (32.83 ± 1.23 vs. 39.47 ± 2.17 m) (Figure 4C). Together, these results suggest that both EPAC1 and EPAC2 are involved in atrial electrophysiology modulation.

**FIGURE 5:**
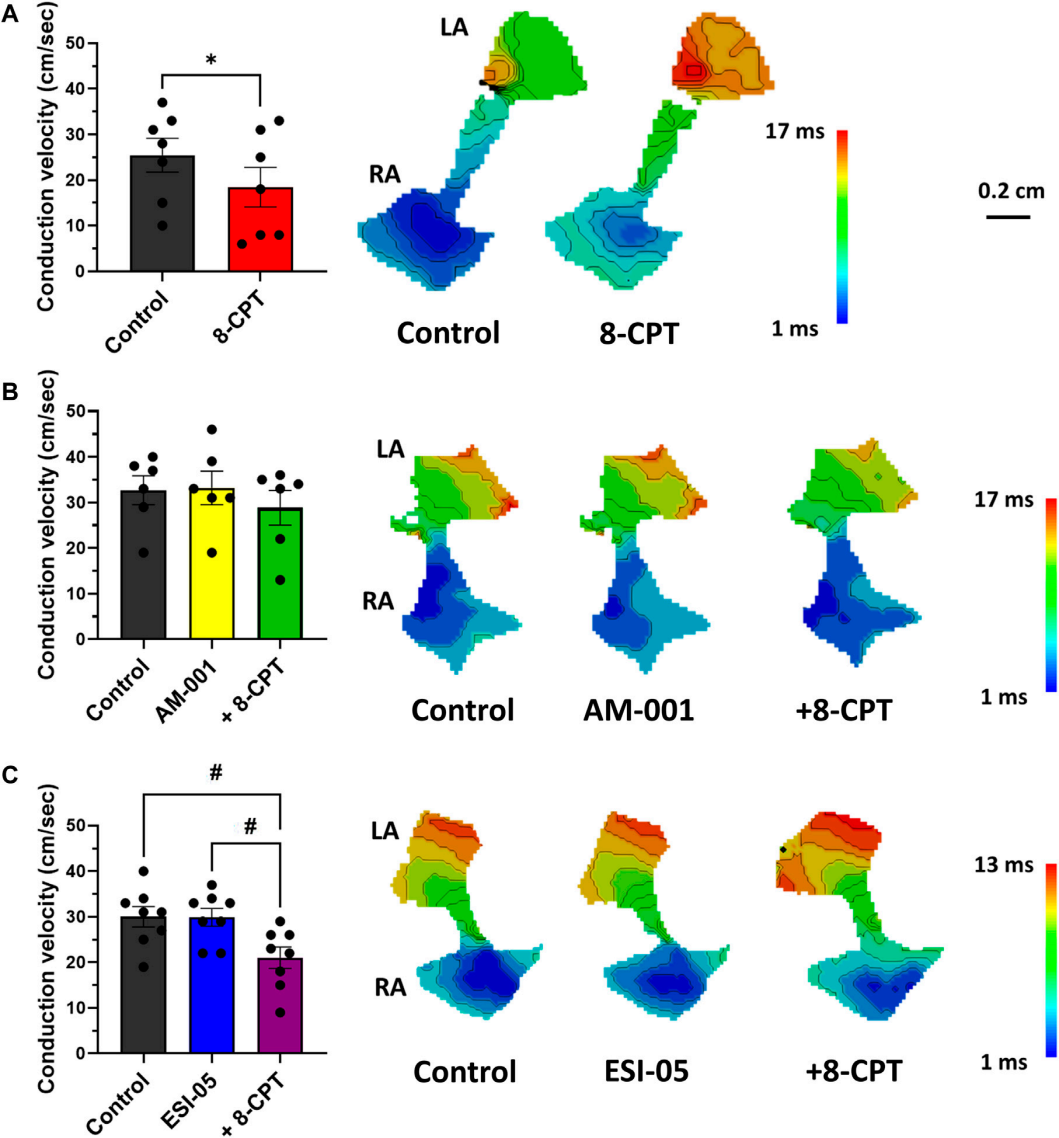
Quantification of conduction velocity (CV) using activation time (AT) maps during 7 Hz pacing. **(A)** Activation of EPAC1&2 leads to a slowing of CV and an increase of total activation time (TAT). (*N* = 7) **(B)** Activation of EPAC2 pathway do not lead to any change in CV and TAT. (*N* = 5) **(C)** Activation of EPAC1 pathway induced a slowing of CV and increased TAT. (*N* = 10) Statistical differences were determined by paired t-tests [**(A)**; *p < 0.05], and by ANOVA with Tukey *post hoc* test and Kruskal Wallis with Dunn *post hoc* test [**(B,C)**; #*p* < 0.05; ##*p* < 0.01].

Optical mapping also facilitated study of AP propagation on across whole atrial tissue surface, and thus calculation of CV. Perfusion of 8-CPT-AM slowed CV by 27% (25.43 ± 3.7 vs. 18.43 ± 4.32 cm/s), and thus we observed an increase in total activation time (Figure 5A). On the contrary, blocking EPAC1 with AM-001 did not modify CV (32.67 ± 3.17 vs. 33.17 ± 3.7 cm/s, Figure 5B). However, AM-001 prevented the effect of 8-CPT-AM on CV, as no significant slowing was observed (32.67 ± 3.17 vs. 28.83 ± 3.80 cm/s). Of particular interest, we did not reveal any effect of EPAC2 pathway in CV since ESI-05 treatment did not significantly change this parameter (30.00 ± 2.24 vs. 29.88 ± 1.95 cm/s) (Figure 5C). However, the addition of 8-CPT-AM to activate EPAC1 in the presence of ESI-05 induced a significant slowing of CV by 30% (30.00 ± 2.24 vs. 21.00 ± 2.34 cm/s). Altogether, these data demonstrate that EPAC1 and not EPAC2 is involved in the modulation of CV.

### 3.3 EPAC pathway modulates cellular atrial electrophysiology

**FIGURE 6:**
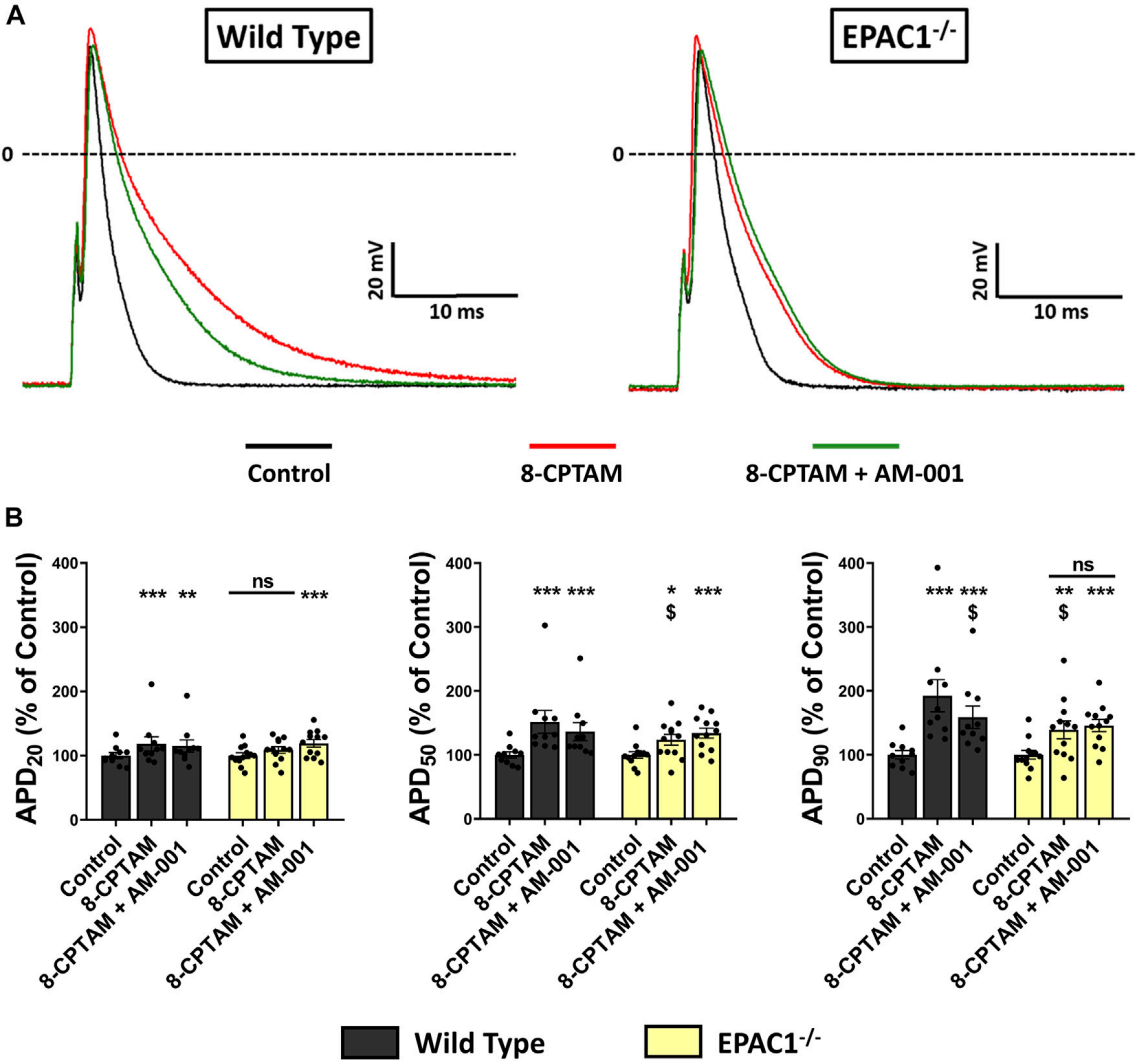
Effect of pharmacological EPAC activation and selective EPAC1 inhibition in APD on WT and EPAC1 deleted atrial myocytes. **(A)** Representative traces of AP in response to EPAC activation and EPAC1 selective inhibition. **(B)** Mean ± SEM of APD20, APD50 and APD90, relative to control (*n* = 10, *N* = 5 for WT and *n* = 12, *N* = 6 for EPAC1−/−). At each percentage of repolarization, EPAC activation by 8-CPT-AM (10 μmol/L) increases APD on WT myocytes, and this effect is partially corrected by EPAC1 selective inhibition. In EPAC1−/− myocytes, effect of 8-CPT-AM is reduced compared to WT and not corrected by AM-001 treatment. Statistical differences were determined by two-way repeated measure ANOVA with Holm-Sidak *post hoc* test and Friedman’s ANOVA on ranks with Dunn’s multiple comparisons post -hoc. *p < 0.05 vs. control, **p < 0.01 vs. control, ***p < 0.001 vs. control. $ is for *p* < 0.05 vs. WT 8-CPTAM condition.

To examine in more detail the role of EPAC1 in atrial electrophysiology, we next measured action potentials using the patch-clamp technique in atrial myocytes isolated from WT and EPAC1^−/−^ mice, before and after superfusion of the EPAC activator 8-CPT-AM (10 μmol/L). In WT atrial myocytes (*n* = 10), as in EPAC1−/− myocytes (*n* = 12), the main effect of EPAC activation was a significant lengthening of APD (Figure 6A) (+92.36 ± 25.22%, *p* < 0.001 and +39.05 ± 14.03%, *p* < 0.01 at 90% of repolarization vs. control, respectively, Figure 6B). However, this lengthening was significantly longer in WT than in EPAC1^−/−^ myocytes (*p* < 0.05). Interestingly, a similar lengthening profile was found at other APDs (at 20% and 50% of repolarization, Figure 6B). 8-CPT-AM had no other effects on AP parameters, except on AP amplitude (Supplementary Table S2). To evaluate whether this lengthening was linked to EPAC2 isoform, atrial myocytes from WT or EPAC1^−/−^ mice were concomitantly superfused with 8-CPT-AM and AM-001. Selective EPAC1 inhibition resulted in a partial but significant correction of the 8-CPT-AM dependent APD lengthening (+58.77 ± 17.49% vs. control at 90% of repolarization, *p* < 0.001 vs. control and *p* < 0.05 vs. 8-CPT-AM condition) in WT atrial myocytes (Figure 6B). In EPAC1^−/−^ atrial myocytes, co-treatment with 8-CPT-AM and AM-001 failed to influence the effect on APD compared to 8-CPT-AM alone (+45.76 ± 9.56% vs. control at 90% of repolarization, *p* < 0.001 vs. control and *p* > 0.1 vs. 8-CPT-AM condition). Interestingly, after EPAC1 selective inhibition with AM-001, 8-CPT-AM induced AP lengthening in WT atrial myocytes which was similar to EPAC1^−/−^ atrial myocytes (*p* = 0.85) (Figure 6B).

**FIGURE 7:**
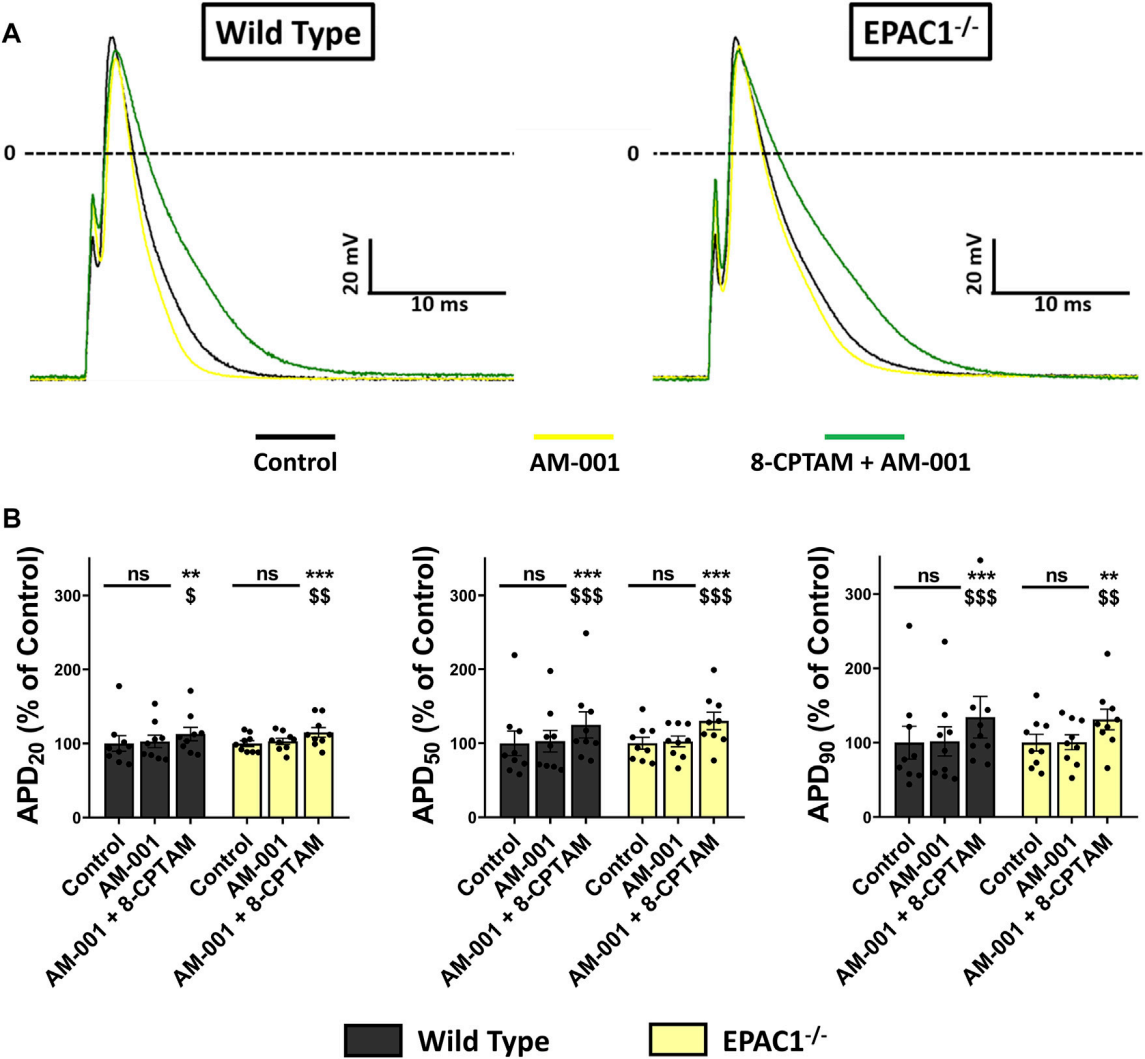
Selective inhibition of EPAC1 by AM-001 treatment reduces 8-CPT-AM dependent APD lengthening in WT myocytes. **(A)** Representative traces of AP in response to EPAC pharmacological activation with 8-CPT-AM, consecutive to pre-treatment with the EPAC1 selective non-competitive inhibitor AM-001 in WT and EPAC1−/− atrial myocytes. **(B)** Mean ± SEM of APD20, APD50 and APD90, relative to control (*n* = 9, *N* = 3 for WT, *n* = 9, *N* = 5 for EPAC1−/−). Treatment with AM-001 (20 μmol/L) seems to have no effect on APD at each percentage of repolarization for each genetic background. EPAC activation with 8-CPT-AM (10 μmol/L) consecutive with this pre-treatment lengthens the APD similarly for WT and EPAC1−/−. Statistical differences were determined by two-way repeated measure ANOVA with Holm-Sidak *post hoc* test. **p < 0.01 vs. control, ***p < 0.001 vs. control. $ is for *p* < 0.05 vs. AM-001 condition, $$ is for *p* < 0.01 vs. AM-001 condition and $$$ is for *p* < 0.001 vs. AM-001 condition.

To determine whether EPAC1 played role in APD modulation at basal level, we superfused AM-001 (20 μmol/L) on WT or EPAC1^−/−^ atrial myocytes and recorded AP parameters (Figure 7A). Inhibition of EPAC1 in basal conditions had no effect on AP parameters in both WT and EPAC1^−/−^ atrial myocytes, except a time-dependent decrease of dV/dtmax (Supplementary Table S3). In addition, superfusion of 8-CPT-AM (10 μmol/L) after pretreatment with AM-001 resulted in APD prolongation both in WT and EPAC1^−/−^ atrial myocytes (*p* < 0.001, *n* = 9 for each background, Figure 7B), suggesting the involvement of EPAC2 in 8-CPT-AM dependent APD increase. Interestingly, after the pretreatment, 8-CPT-AM dependent APD prolongation was similar in both WT and EPAC1^−/−^ mice (+34.23 ± 27.87% and +31.23 ± 13.95% vs. control at 90% of repolarization, respectively, *p* = 0.73). Taken together, these results show that EPAC1 and EPAC2 isoforms are both involved in the effect of 8-CPT-AM in the APD prolongation in mouse atrial myocytes.

## 4 Discussion

Our study provides the first comprehensive evaluation of EPAC involvement in atrial electrophysiology and AF susceptibility. We show that EPAC1 and EPAC2 signalling pathways increase APD in atria at the tissue and cellular levels. However, only EPAC1 isoform is involved in AF genesis in the whole animal. Key findings demonstrate that reduced CV may represent a substrate for AF during EPAC activation, and that AM-001, an EPAC-1 selective pharmacological inhibitor, prevents AF.

### 4.1 Link to previous studies

Prior work on EPAC signaling pathways in cardiac pathophysiology have focused on the ventricle. EPAC activates pro-hypertrophic pathways leading to cardiomyocyte hypertrophy (Morel et al., 2005). Other deleterious effects of EPAC are ventricular arrhythmias, first described *in vitro*, and confirmed in transgenic mice for EPAC1 and EPAC2 (Laudette et al., 2018). At the cellular level, EPAC regulates AP lengthening in rat ventricular cardiomyocytes (Brette et al., 2013), a process which is correlated with the genesis of arrhythmia by predisposing cardiomyocytes to early after depolarization and dispersion of repolarization (Nattel et al., 2007). In addition, EPAC activation increases Ca^2+^ spark frequency (Pereira et al., 2007). Chronic activation of EPAC influences the expression level of proarrhythmic channels including the slow delayed-rectifier potassium current (IKs) subunit potassium voltage-gated channel (KCN) and transient receptor potential canonical 3 and 4 channels (TRPC3/4) (Aflaki et al., 2014; Dominguez-Rodriguez et al., 2015). At the atrial level little is known. Acute EPAC activation inhibits the sodium current *via* ryanodine receptor type 2 activation. This decrease in Na^+^ current is associated with a reduction in maximum AP upstroke rate (Valli et al., 2018). In our study, we did not find any significant change in AP upstroke rate (Supplementary Table S2). The discrepancy can be due to the difference of genetic backgrounds of the mice and/or age of the animals. However, our data are consistent with recent studies showing an involvement of EPAC1 in the development of atrial and ventricular arrhythmias in mice (Prajapati et al., 2019; Zhang et al., 2019).

### 4.2 Atrial electrophysiology

As described in ventricular cardiomyocytes, EPAC activation in atria lead to a lengthening of action potential at the tissue and cellular level. Our results demonstrated that both EPAC1 and EPAC2 isoforms are involved in the APD changes. In ventricular myocytes, APD lengthening is due to a decrease in sustained potassium current (Brette et al., 2013). Ion channels underlying AP are different in atria compared to ventricles (Hatem et al., 2010). The main difference is in potassium channels, albeit sustained potassium current is also present in atria. CV is an important parameter for atrial cardiac arrhythmia (Weiss et al., 2005). Our results from optical mapping experiments showed that CV is decreased upon EPAC activation. Ion channels regulating CV are connexins and sodium channels (Campbell et al., 2014). Given our patch-clamp data, it seems likely that the decrease in CV is due to changes in connexin properties. Further studies are needed to determine which ion channels are modulated by EPAC in atria, including connexin.

### 4.3 EPAC and AF susceptibility

Our results showed that AF susceptibility was increased when EPAC was activated by 8-CPT. It seems likely that an AF episode is triggered by reduction in CV. To dissect which isoform was important in AF susceptibility, we used KO mice and our data showed that AF susceptibility was decreased in EPAC1 KO mice. These results are consistent with a study focusing on EPAC1-dependent phospholamban phosphorylation which also show that EPAC1 deficiency resulted in reduced susceptibility to AF (Okumura et al., 2014). In contrast, in EPAC2 KO mice AF susceptibility was similar to WT animals. The implication of EPAC1, and not EPAC2 is confirmed by our pharmacological approach, where AM-001, a specific EPAC1 inhibitor prevented AF. These results are consistent with a study showing that acute effects of CE3F4 treatment, an EPAC1 selective inhibitor (Courilleau et al., 2012; Courilleau et al., 2013), shorten the duration of AF in mice (Prajapati et al., 2019).

### 4.4 Potential relevance of AM-001

Until recently, the only EPAC1 selective inhibitor identified was CE3F4, a tetrahydroquinoline analogue, but its low bioavailability prevented its biological action *in vivo*, thereby excluding its use in large animals (Courilleau et al., 2012; Courilleau et al., 2013; Laudette et al., 2019). Another non-cyclic nucleotide small molecule designed as ESI-09 displays a favorable pharmacological/toxicological profile when administrated *in vivo* to mice but this compound does not discriminate between EPAC1 and EPAC2 isoforms (Almahariq et al., 2013). In the current study, we used AM-001, an EPAC1 non-competitive inhibitor, with *in vivo* application (Laudette et al., 2019). Our data demonstrate that AM-001 prevents AF in mice. Further studies are needed to confirm this finding in other mammal species.

### 4.5 Study limitations

There are several limitations to our study: First, mouse as an animal model to study AF is still controversial (Fu et al., 2022). However, numerous studies used mice to investigate the mechanisms involved in the initiation of AF, as reviewed in (Riley et al., 2012; Clauss et al., 2019; Blackwell et al., 2022). In addition, mouse models of AF may be useful to uncover novel mechanisms of AF. This is well illustrated with the studies demonstrating that abnormal ryanodine receptor Ca^2+^ release *via* CaMKII overactivation is directly responsible for the progression of paroxysmal AF to more persistent forms (Li et al., 2014; Mesubi and Anderson, 2016; Landstrom et al., 2017). A second limitation of our study concerns the data generated from transesophageal stimulation experiments. In the majority of results, we observed a trend which did not reach statistically significance. This was probably due to the large variability in this approach. Indeed, even if transesophageal stimulation protocol for the induction of AF in mice was carefully evaluated more than 20 years ago (Schrickel et al., 2002), data showed that AF inducibility in control mice could range from 0% to more than 70% indicating that optimization of this technique is still required (Murphy et al., 2022). Power analysis from our experiments indicates than more 30 mice in each condition should have been used, but this conflicts with our 3R ambitions. Another limitation would be the off-target effects of the drugs used but the specificity of 8-CPT-AM towards EPAC had been previously demonstrated in double KO mice for EPAC1 and EPAC2 (Pereira et al., 2013). Finally, the mechanistic link between EPAC activation, APD prolongation and the decreased CV in atria is unknown and requires extensive work in subsequent studies.

## 5 Conclusion

In summary, our data reveal that EPAC1 and EPAC2 modulates atrial electrophysiology. EPAC1 is involved in AF genesis. AM-001, an EPAC1 inhibitory compound may be a potential therapeutic drug candidate to prevent AF.

## Data Availability

The raw data supporting the conclusion of this article will be made available by the authors, without undue reservation.
