# Correction: Inhibition of EPAC1 signaling pathway alters atrial electrophysiology and prevents atrial fibrillation

**DOI:** 10.3389/fphys.2025.1672433

**Published:** 2025-08-14

**Authors:** Bastien Guillot, Arthur Boileve, Richard Walton, Alexandre Harfoush, Caroline Conte, Yannis Sainte-Marie, Sabine Charron, Olivier Bernus, Alice Recalde, Laurent Sallé, Fabien Brette, Frank Lezoualc’h

**Affiliations:** ^1^ IHU LIRYC -CRCTB U1045, Pessac, France; ^2^ INSERM U1045 -Université de Bordeaux, Bordeaux, France; ^3^ UR 4650 PSIR, GIP Cyceron, Caen, France; ^4^ Université de Caen-Normandie, Caen, France; ^5^ Université de Toulouse-Paul Sabatier, Toulouse, France; ^6^ Institut des maladies métaboliques et cardiovasculaires, INSERM UMR-1297, Toulouse, France; ^7^ PhyMedExp, INSERM U1046, CNRS 9412, Université de Montpellier, Montpellier, France

**Keywords:** EPAC, atrial fibrillation, optical mapping, cardiomyocytes, action potential

In the published article, there was a mistake in the **Funding** statement. The **funding** statement for the ANR ELECTRO was displayed as “ANR-19-CE17-0010 (ELECTRO)”. The correct statement appears below.

