# Supplementary material for: Inhibition of EPAC1 signaling pathway alters atrial electrophysiology and prevents atrial fibrillation
Source: Front Physiol. 2023 Feb 22;14:1120336. doi: 10.3389/fphys.2023.1120336 (PMC9992743; doi:10.3389/fphys.2023.1120336)
Supplement: Supplementary file 1 [file DataSheet1.pdf]

Supplementary Material

Inhibition of EPAC1 signaling Pathway Alters Atrial Electrophysiology and Prevents Atrial Fibrillation

Bastien Guillot, Arthur Boileve, Richard Walton, Alexandre Harfoush, Caroline Conte, Yannis Sainte-Marie, Sabine Charron, Olivier Bernus, Alice Recalde, Laurent Sallé, Fabien Brette*, Frank Lezoualc'h

*** Correspondence:** Fabien Brette: fabien.brette@inserm.fr

1 Supplementary Figures and Tables

1.1 Supplementary Figures

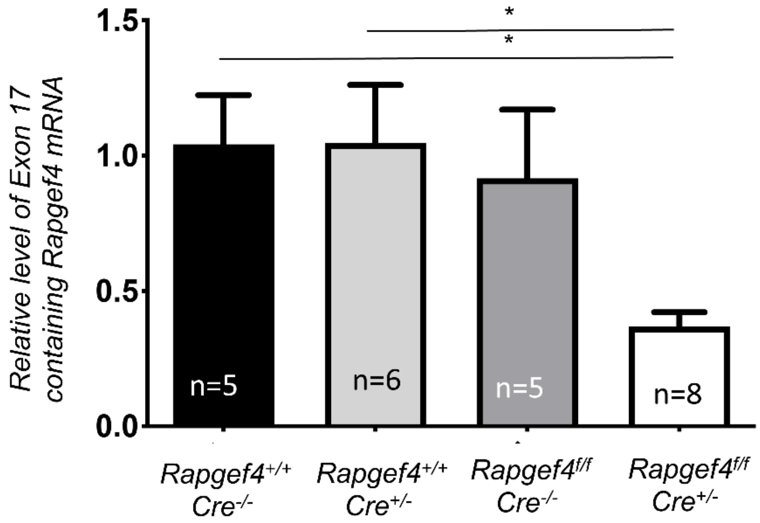

Supplementary Figure 1.

Quantitative RT-PCR analysis of Rapgef4 mRNA expression in left ventricules from mice Rapgef4+/+ and Rapgef4f/f expressing (Cre+/-) or not (Cre-/-) the Cre recombinase using two couples of primers (Ex3F TGTTAAAGTGTCTGAGACCAGCA with Ex4R AAAGGCTGTCCCAATTCCCAG and Ex17F TTATGCCAAATACCCAGCTTTG with Ex18R CTCTTGTTGTTGAGGGCATAATC) . Rapgef4 exon 17 was quantified relatively to Rapgef4 mRNA level.(reference gene = RPL13)

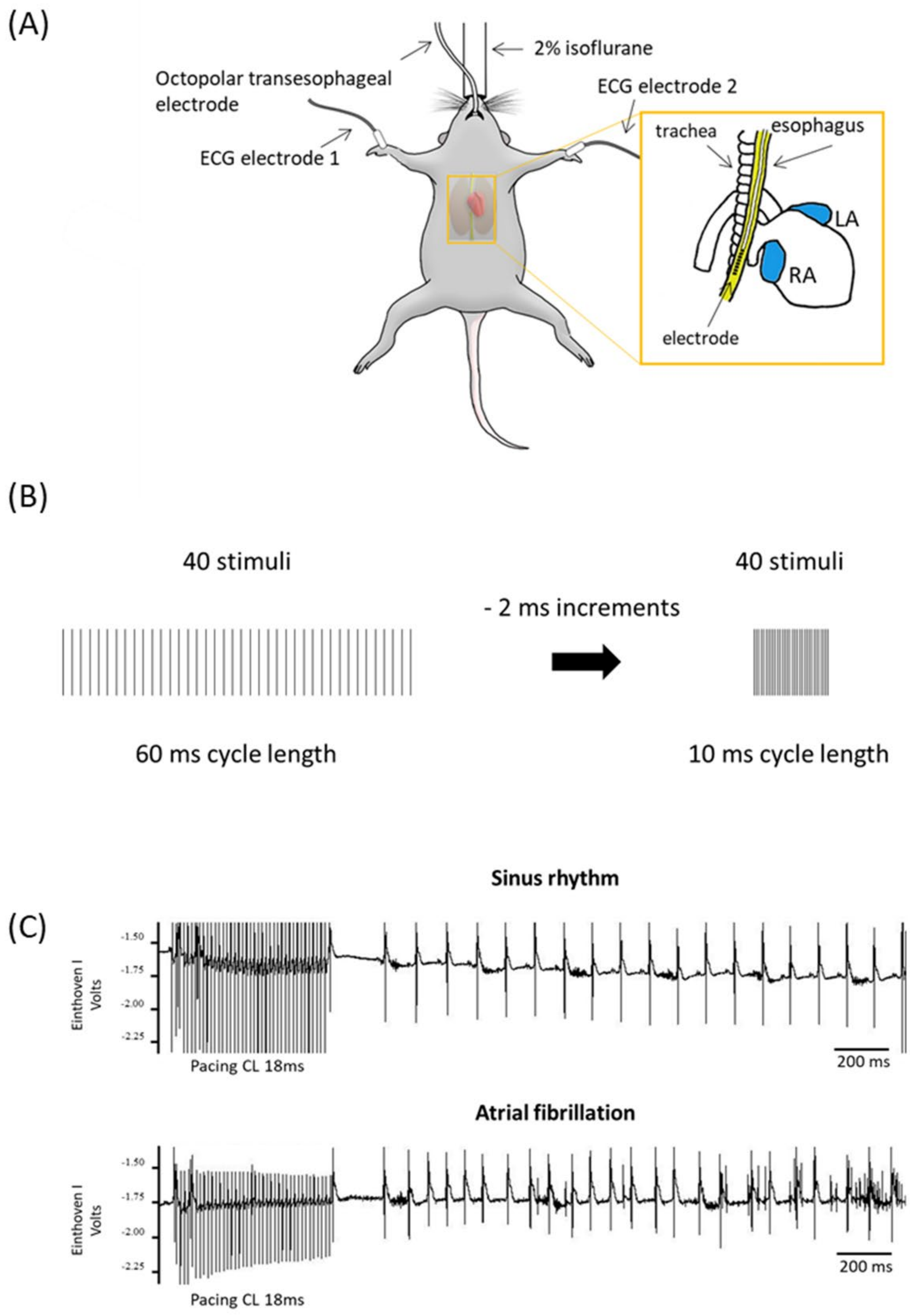

Supplementary Figure 2.

Assessment of AF inducibility and probability by rapid transesophageal stimulation. (A) Schematic representation of the TS system by the introduction of an octopolar catheter and ECG recording. (B) Pacing protocol used to evaluate AF inducibility and probability. (C) Representative ECG traces of sinus rhythm and AF episode.

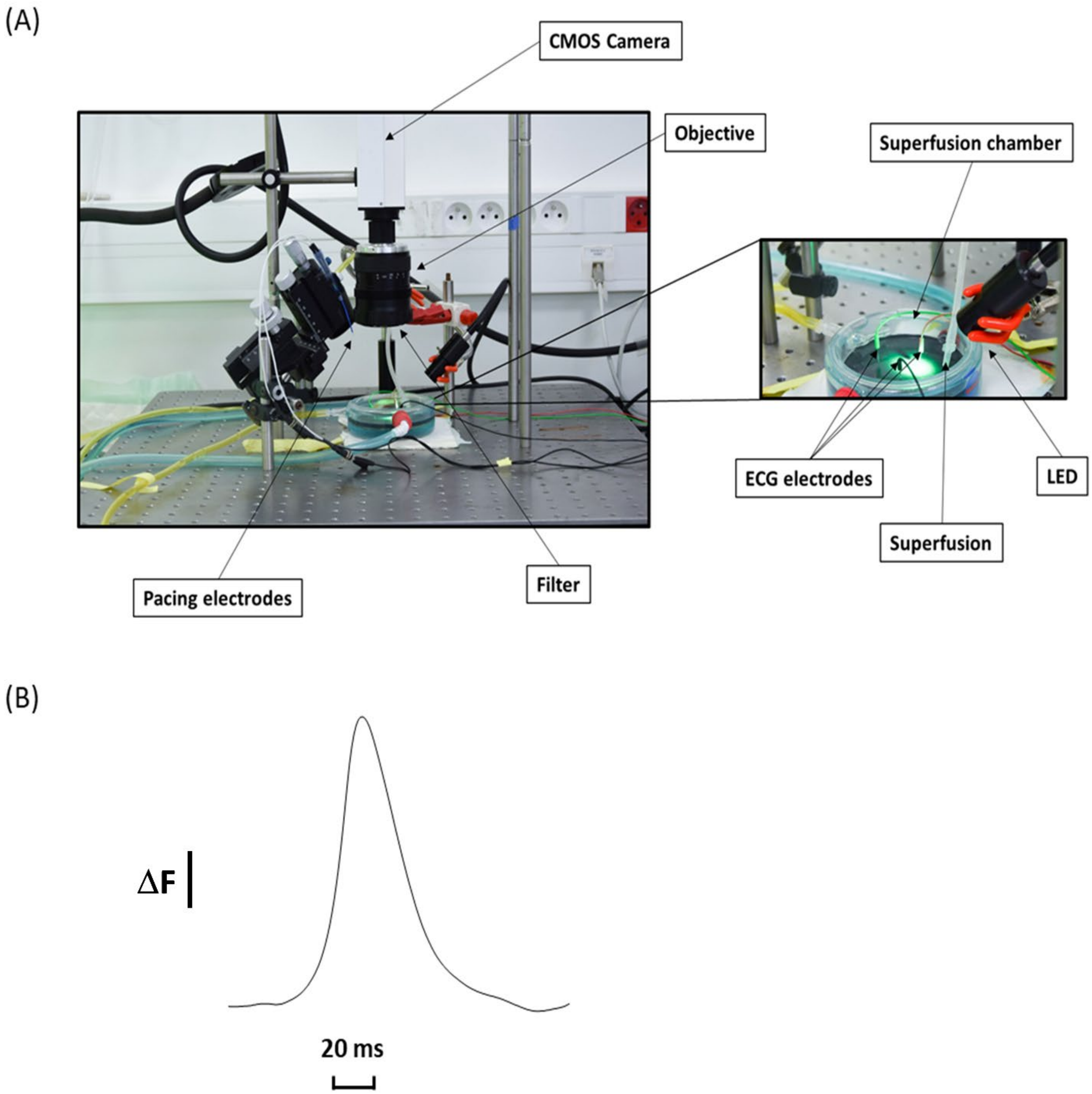

Supplementary Figure 3.

Electrophysiological properties of mice atria were studied using optical mapping set-up. (A) Optical mapping set-up is composed by a CMOS camera, an objective, a LED, and a perfusion and pacing device. (B) Representation of an Optical Action Potential (OAP) recorded thanks to the optical mapping set-up, and used to study electrophysiological properties of mice atria.

(A)

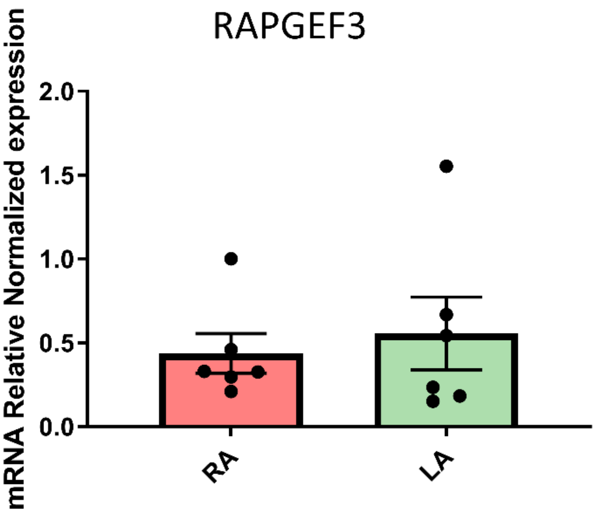

(B)

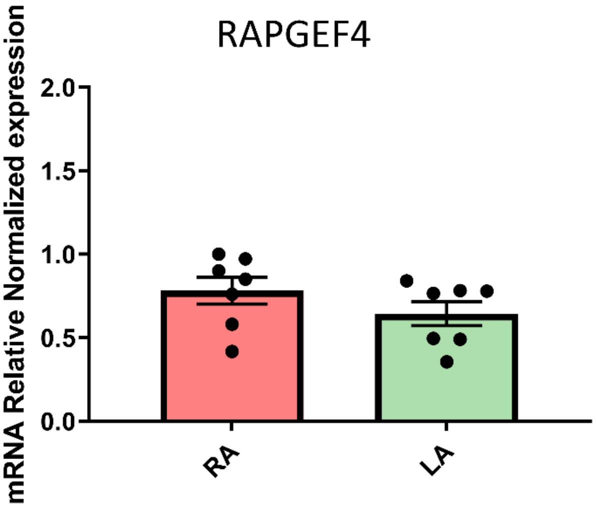

Supplementary Figure 4.

Relative expression of EPAC genes in mouse atria. (A) EPAC1 mRNA (RAPGEF3) expression in mouse right atria (RA) and left atria (LA). (B) EPAC2 mRNA (RAPGEF4) expression in mouse RA and LA. N=7.

1.2 Supplementary Tables

Supplementary Table 1.

Table S1. Heart size and cardiac function

	**Cnt (n)**	**Epac2 cKO (n)**
**Heart /Tibia (mg/cm)**	64.8 +/- 1.4 (11)	63.8 +/- 1.6 (16)
**Left Ventricle/Tibia (mg/cm)**	49.7 +/- 1.1 (11)	48.7 +/- 1.4 (16)
**Left Atria (mg)**	2.8 +/- 0.1 (11)	2.7 +/- 0.2 (16)
**Heart Rate (bpm)**	507 +/- 17 (7)	443 +/- 12 (14) *
**LVAW,d (mm)**	0.83 +/- 0.05 (7)	0.79 +/- 0.03 (14)
**LVPW, d (mm)**	0.73 +/- 0.02 (7)	0.63 +/- 0.02 (14) *
**LVID, d (mm)**	4.32 +/- 0.05 (7)	4.32 +/- 0.06 (10)
**FS (%)**	24.1 +/- 0.9 (7)	25.1 +/- 1.2 (14)
**EF (%)**	41.8 +/- 0.9 (7)	43.8 +/- 1.4 (14)

Abbreviations : LVAW,d : left ventricle end-diastolic anterior wall thickness ; LVPW,d : left ventricle end-diastolic posterior wall thickness ; LVID,d : left ventricle internal diastolic diameter ; FS : fractionnal shortening ; EF : ejection fraction

Data are mean+/-sem. * P<0.05 by unpaired t-test with Welch's correction

Supplementary Table 2.

Table S2. Effect of 8-CPTAM dependent Epac activation and Epac1 selective inhibition on AP parameters in Wild Type and Epac1 depleted atrial myocytes

	WILD TYPE			EPAC1$^{-/-}$		
	Control	8-CPTAM	AM-001 + 8-CPTAM	Control	8-CPTAM	AM-001 + 8-CPTAM
Holding Membrane Potential (mV)	-81.0 ± 1.7	-81.8 ± 1.6	-82.6 ± 1.8	-80.2 ± 1.6	-81.1 ± 1.8	-82.5 ± 1.7
Amplitude (mV)	109.6 ± 3.9	119.3 ± 2.8*	115.4 ± 2.9	110.3 ± 3.5	117.0 ± 2.8	113.8 ± 3.0
dV/dTmax (mV/ms)	150.2 ± 14.4	148.7 ± 13.1	130.9 ± 12.9	157.3 ± 15.4	156.3 ± 15.3	133.8 ± 11.2
APD20 (ms)	2.8 ± 0.1	3.3 ± 0.3***	3.2 ± 0.2**	2.6 ± 0.1	2.8 ± 0.1	3.1 ± 0.2***
APD50 (ms)	3.8 ± 0.2	5.7 ±0.7***	5.1 ± 0.5***	3.7 ± 0.2	4.5 ± 0.3*** $	4.9 ± 0.3***
APD90 (ms)	6.4 ± 0.4	12.3 ± 1.6***	10.2 ± 1.1*** $	6.6 ± 0.4	9.2 ± 0.9** $	9.7 ± 0.6***

Values are mean ± SEM for 10 cells on WT mice and 12 cells on Epac1$^{-/-}$ mice.

* indicates *P*<0.05 *vs* control, ** indicates *P*<0.01 *vs* control and *** indicates *P*<0.001 *vs* control.

$ indicates *P*<0.05 *vs* WT 8-CPTAM condition.

Supplementary Table 3.

Table S3. Effect of 8-CPTAM dependent Epac activation on AP parameters in Wild Type and Epac1 depleted atrial myocytes consecutively to pre-treatment with the EPAC1 selective inhibitor AM-001

	WILD TYPE			EPAC1$^{-/-}$		
	**Control**	**AM-001**	**AM-001 + 8-CPTAM**	**Control**	**AM-001**	**AM-001 + 8-CPTAM**
Holding Membrane Potential (mV)	-83.1 ± 2.5	-84.6 ± 2.4	-84.1 ± 2.6	-82.7 ± 1.1	-84.1 ± 1.4	-84.1 ± 1.5
Amplitude (mV)	115.8 ± 3.9	119.7 ± 2.9	119.7 ± 3.4	120.6 ± 1.7	122.8 ± 1.8	120.2 ± 2.9
dV/dTmax (mV/ms)	164.8 ± 9.5	141.1 ± 10.4**	135.0 ± 9.7***	169.3 ± 10.5	156.8 ± 11.9	139.2 ± 14.3*** $
APD20 (ms)	3.4 ± 0.4	3.5 ± 0.3	3.9 ± 0.3** $	3.4 ± 0.1	3.5 ± 0.1	4.0 ± 0.2*** $$
APD50 (ms)	5.8 ± 1.0	5.9 ± 0.8	7.2 ± 1.0*** $$$	5.5 ± 0.4	5.7 ± 0.4	7.2 ± 0.7*** $$$
APD90 (ms)	12.2 ± 2.7	12.5 ± 2.4	16.4 ± 3.4*** $$$	11.7 ± 1.3	11.8 ±1.2	15.3 ± 1.6** $$

Values are mean ± SEM for 9 cells for each condition.

** indicates *P*<0.01 *vs* control, *** indicates *P*<0.001 *vs* control.

$ indicates *P*<0.05 *vs* AM-001 conditions, $$ indicates *P*<0.01 *vs* AM-001 condition and $$$ indicates *P*<0.001 *vs* AM-001 condition.
